# Peroneal Nerve Dysfunction in Patients With Clubfoot Deformity: Evaluation of Clinical Presentation and Treatment

**DOI:** 10.7759/cureus.72165

**Published:** 2024-10-22

**Authors:** Nasir Baig, Mohammed A Alzahrani, Ahmed A Abdulkader, Renad A Alshaer, Rana S Alghmdi

**Affiliations:** 1 Orthopedics, Alhada Armed Forces Hospital, Taif, SAU; 2 Medicine, Taif University, Taif, SAU

**Keywords:** congenital talipes equinovarus (ctev), dysfunction, nerve, peroneal, tibialis posterior tendon transfer

## Abstract

The congenital foot drop associated with congenital talipes equinovarus (CTEV) deformity secondary to common peroneal nerve injury is rare. Moreover, the foot drop may even be completely overlooked when associated with clubfoot due to its similar appearance. We report a case of peroneal nerve dysfunction in a child with a late presentation of congenital clubfoot who underwent tibialis posterior transfer to improve mobility and function.

## Introduction

Congenital talipes equinovarus (CTEV), or clubfoot, is a common developmental disorder characterized by the foot being fixed in adduction, supination, and varus-angled inward, rotated outward, and pointed downward. Its prevalence ranges from 0.5 to 2 per 1,000 live births in low- and middle-income countries, with about half of cases being bilateral and most unilateral cases being right-sided. CTEV can be idiopathic or syndromic. Approximately 80% of cases are idiopathic, while the rest are associated with other malformations or genetic disorders [1].

The cause of CTEV is unclear, but there is evidence of a genetic component. Approximately 24.4% of isolated cases have a family history, and studies show higher concordance in monozygotic twins (33%) compared to dizygotic twins (3%), with an estimated heritability of approximately 30%. However, the inheritance pattern does not follow typical Mendelian genetics, as seen in the unusual male-to-female ratio of 2.0-2.5:1 [2].

Syndromic CTEV, which can resemble idiopathic CTEV, is often linked to neurological or neuromuscular disorders and fetal abnormalities. As a result, idiopathic and syndromic CTEV differ in clinical features, development mechanisms, and treatment options. Here, we report a unique case of CTEV complicated with peroneal nerve dysfunction of idiopathic drop foot with subsequent tibialis posterior tendon transfer, achieving significant improvement [2].

## Case presentation

CTEV presented late at one year and five months in a boy who was born full-term by cesarean delivery. The prenatal, natal, and postnatal history was unremarkable, with no dysmorphic features or congenital issues noted. Two clinical signs to differentiate between foot drop and CTEV are the prominence of the lateral metatarsal heads with dimpling of the intermetatarsal spaces and drop toe signs; however, these were not clear in our case.

The patient underwent serial casting, with about four casts applied, followed by the use of a foot abduction orthosis. At the age of three years and nine months, the patient presented to the clinic. He could walk independently but on the lateral border of the foot with a steppage gait and dynamic supination. A clinical picture of a foot drop was noticed in the affected limb. The flaccid type of equinus foot could be dorsiflexed to more than 30 degrees, indicating a flexible midfoot cavus deformity with no forefoot adduction.

**Figure 1:**
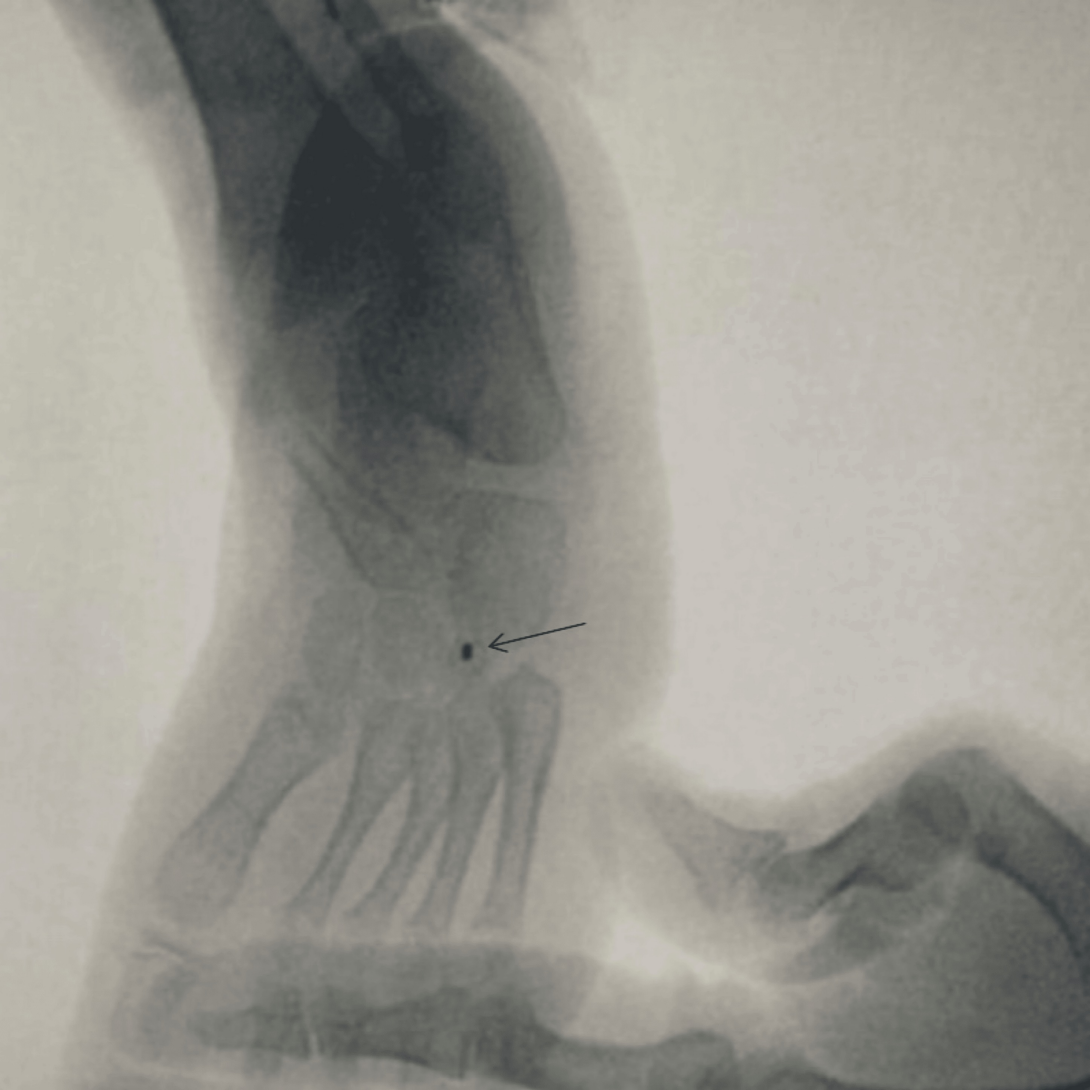
Intraoperative X-ray of the left foot during posterior tibial tendon transfer to the lateral cuneiform.

The power of ankle dorsiflexion and eversion was 0/5, while the power of inversion and plantarflexion was 4-5/5. The patient was followed up at a pediatric neurology clinic, where an MRI of the lower limbs was ordered, revealing no abnormal findings. Nerve conduction studies (NCS) and electromyography (EMG) showed a partial axonal lesion of the common peroneal nerve with poor evidence of regeneration, but the left tibial nerve and left tibialis posterior muscle were intact. The patient underwent a tibialis posterior tendon transfer to the dorsum of the foot (lateral cuneiform), as shown in Figure 1.

**Figure 2:**
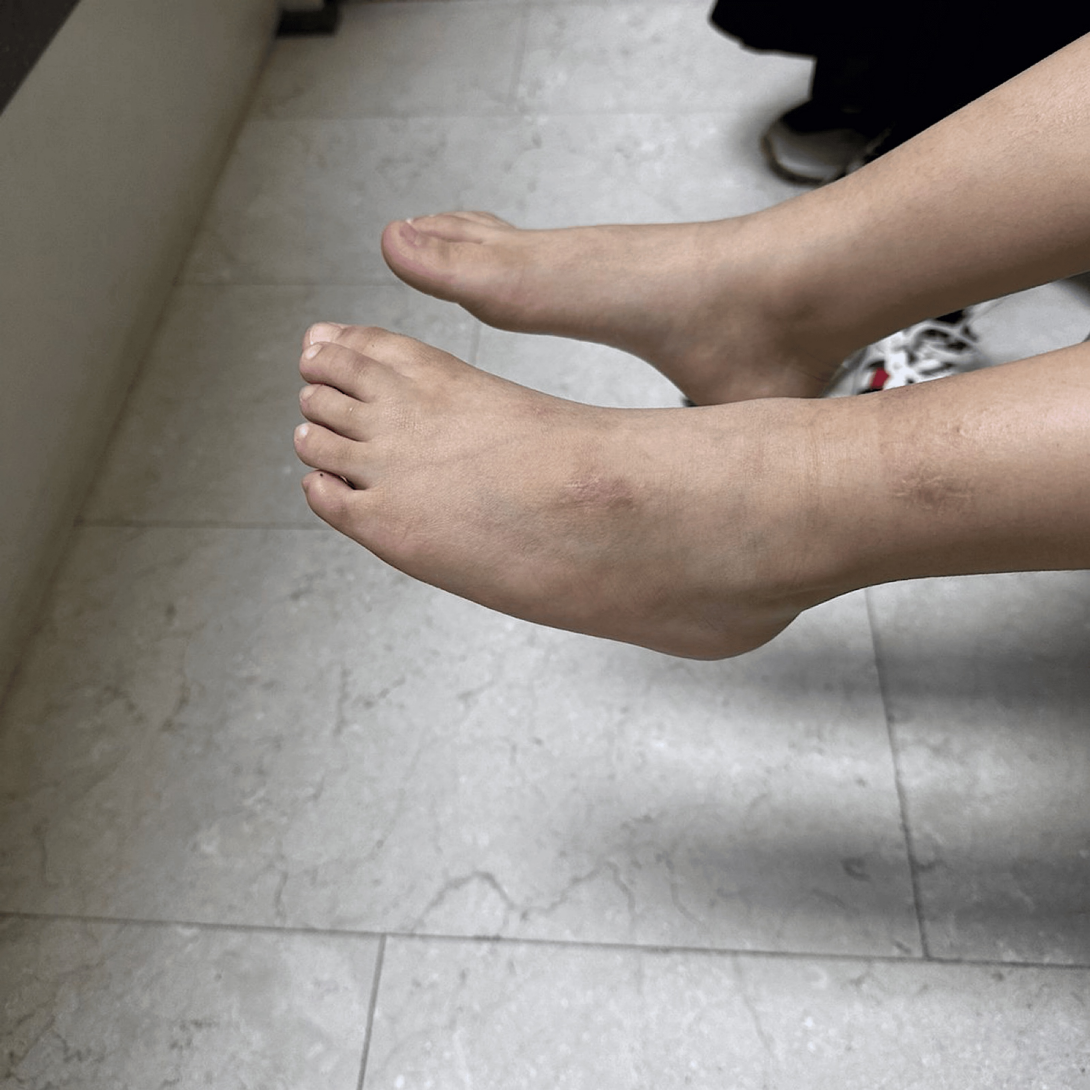
Left foot with gravity plantar flexion.

**Figure 3:**
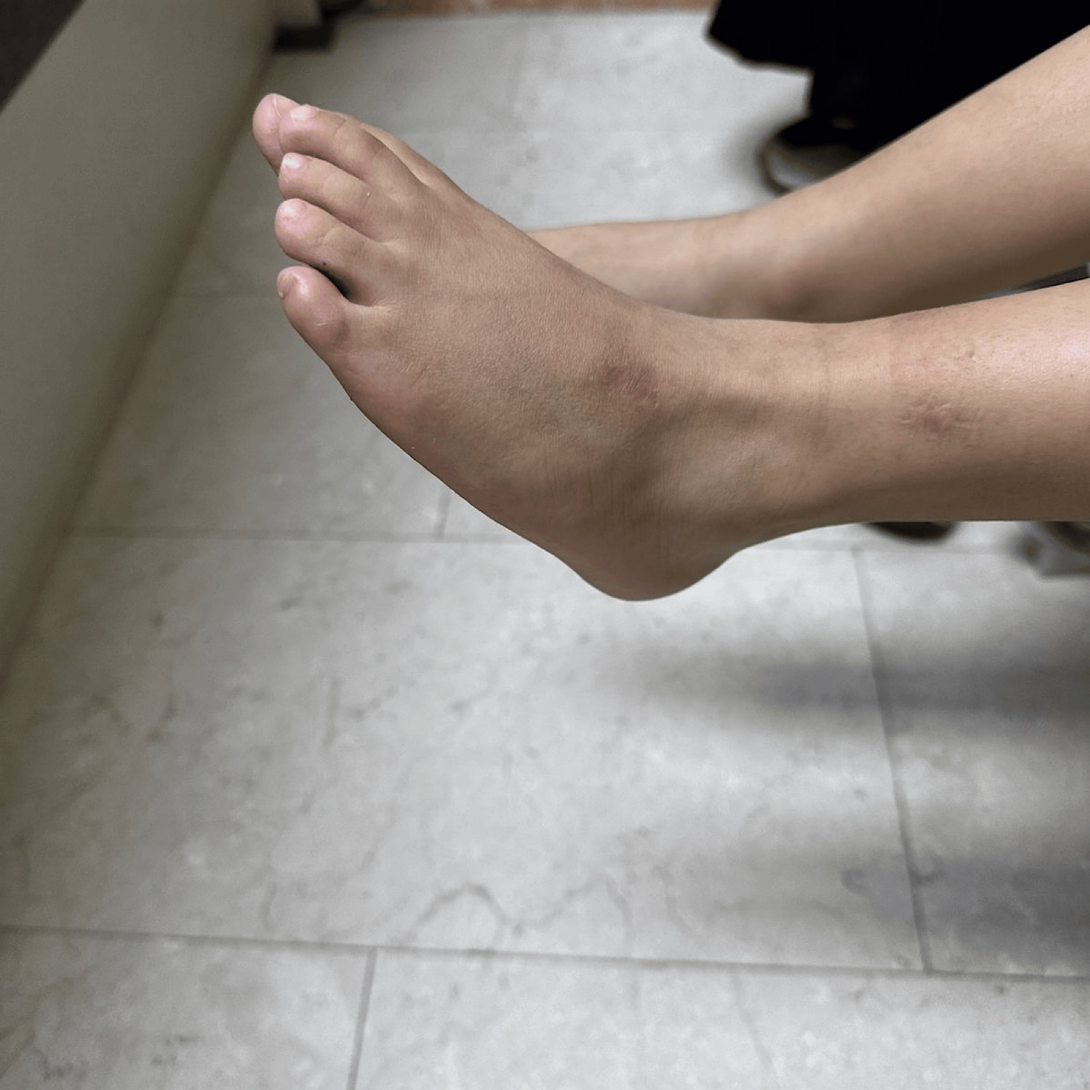
Left foot with active dorsiflexion.

At the age of four years, the patient demonstrated improvement in ankle dorsiflexion (3-4/5), a reduction in steppage gait, and excellent clearance during the swing phase (Figures 2, 3).

## Discussion

Idiopathic congenital clubfoot is a widespread and severe birth defect affecting the musculoskeletal system worldwide. This condition typically persists into adulthood, leading to substantial impairments in function and overall quality of life. The Ponseti method, which includes a series of casts, Achilles tenotomy, and the use of braces, is an established treatment strategy for managing this condition [3].

The common peroneal nerve is vulnerable to compression and injury as it wraps around the fibular neck. Foot drop refers to the inability to dorsiflex the foot, resulting from weakness in the muscles responsible for this movement. This condition can arise from either weakness in the dorsiflexor muscles or damage to the common fibular nerve, which innervates these muscles. Congenital foot drop in neonates is rare and usually linked to underlying neurological, muscular, or structural issues, such as the deformity known as talipes equinovarus. Common causes of peroneal neuropraxia or other mononeuropathies include obstetric complications, such as birth trauma or abnormal fetal positioning, like breech or transverse lie. Mononeuropathies in newborns are infrequent and generally result from birth-related trauma or in-utero compression.

The most common peripheral nerve injuries in newborns involve the brachial plexus and facial nerve [4]. Congenital foot drop from common peroneal nerve injury is relatively uncommon in newborns and often reflects abnormalities related to lower motor neuron lesions of the common peroneal nerve, sciatic nerve, or, less frequently, upper motor neuron lesions affecting the spinal cord or motor cortex. It can also result from structural defects in the periarticular bones and ligaments [4]. Isolated congenital foot drop in newborns due to common peroneal nerve palsy is extremely rare.

A retrospective study of 658 patients treated for clubfoot revealed cases of complete unilateral common peroneal nerve dysfunction associated with congenital clubfoot [7]. Patients received treatment with Ponseti casts, Achilles tenotomy, and foot abduction bracing. In every case, the diagnosis of complete peroneal nerve dysfunction was confirmed through nerve conduction velocity studies. After full-time bracing, the patients were provided with an insole polyethylene molded ankle-foot orthosis. Three patients also underwent tibialis posterior tendon transfer to enhance foot dorsiflexor strength [7].

In the neonatal population, foot drop is primarily attributed to compression, trauma, or entrapment of the common peroneal nerve, often due to constriction bands. While isolated peroneal nerve palsies caused by pressure or compression at the fibular head generally have a favorable prognosis, there is a risk of exacerbating the common peroneal nerve palsy with repeated cast pressure in the context of CTEV treatment. Common peroneal nerve neuropathy due to cast pressure typically recovers fully over time without specific intervention. In our case, however, common peroneal nerve dysfunction persisted despite the use of Dennis Browne splints until age four, indicating that the dysfunction was not related to cast pressure.

Uncommon causes of foot drop include ischemic necrosis of the gluteal region affecting the sciatic nerve after umbilical artery catheterization and Group B streptococcus osteomyelitis. However, in these cases, foot drop was not isolated but accompanied by additional localized signs. Literature reports various morphological changes associated with clubfoot and common peroneal nerve dysfunction, such as prominence of the lateral metatarsal heads, dimpling of intermetatarsal spaces, and drop toe signs; however, these features were not evident in our case. Investigations into foot drop should be guided by clinical findings to exclude structural issues involving bones, nerves, and tendons around the ankle joint. Imaging of the brain and spine may be considered if clinically warranted. In neonates, it is important to account for gestation-dependent motor nerve conduction velocities, which can affect interpretation. Additionally, performing NCS in neonates can be technically difficult due to their limited tolerance for discomfort, often necessitating sedation to obtain accurate results.

Some studies emphasize the importance of initially performing a detailed neurological examination for all clubfoot patients to detect the drop toe sign. However, this sign may also be present in individuals with other types of neurogenic clubfoot. While recognizing the drop toe sign typically does not change the management needed to correct the deformity, it does signal the need for further evaluation [5,6]. In another study involving three patients aged above five years, the clinical assessment revealed limb length shortening, specifically in the tibia, without any femur shortening. Calf muscle atrophy was observed, and there were no instances of relapse or residual deformities during or after the bracing period. These patients exhibited prominent metatarsal heads of the fifth, fourth, and/or third metatarsals, along with dimple lesions, on the dorsal aspect.

Additionally, increased intermetatarsal webbing of the lateral three or four toes, without syndactyly, was consistently noted, and these features became more prominent with age. In the presented cases of clubfoot deformity, the medical protocol encompassed Ponseti casting for deformity correction, Achilles tenotomy for persistent issues, and the utilization of foot abduction braces following the standard Ponseti protocol. NCS during the bracing period were conducted to confirm the diagnosis of a complete common peroneal nerve dysfunction. Subsequently, insole polyethylene molded ankle-foot orthosis was provided to address foot drop during the awake period.

Consequently, they underwent further interventions, including tibialis posterior transfer for foot dorsum correction to the lateral cuneiform in these patients, aiming for active ankle dorsiflexion until the neutral position (0°). However, only partial correction of the foot drop gait pattern was achieved. Consequently, both the ankle-foot orthosis and foot abduction brace were discontinued for these three patients, while the insole foot drop support continued for ongoing management. However, they believe that the utilization of posterior tibial tendon transfer to supplement ankle dorsiflexor function may not lead to the desired improvement in gait.

Comparing our case with previous cases, there are no peripheral or central causes, dysmorphic features, or congenital issues in either instance. Common perineal nerve dysfunction is observed in our case, similar to previous cases. However, distinctions arise as dimple lesions were present in prominent metatarsal heads of the fifth, fourth, and/or third on the dorsal aspect in previous cases, whereas our case showed no dimple lesions. The peripheral MRI revealed no abnormal findings, but NCS indicated partial peroneal nerve dysfunction in our case, in contrast to previous cases in which complete peroneal nerve dysfunction was observed.

We found significant improvement after tibialis posterior tendon transfer to lateral cuneiform, especially in steppage gait, excellent clearance during the swing phase, and power of ankle dorsiflexion by 3-4/5, which was 0/5 before tibialis posterior tendon transfer. Ultimately, the transfer of the tibialis posterior to the dorsum of the foot is considered necessary for these patients at a suitable age. However, even if the tendon transfer is executed correctly and enables active ankle dorsiflexion post-transfer, the enhancement in foot drop gait might only be partial. Challenges such as diminished ankle dorsiflexion range of motion, severe calf muscle atrophy, and variations in the tibialis posterior muscle excursion compared to the tibialis anterior could diminish the success of the transfer. It is crucial to discuss these potential outcomes with parents before proceeding with the procedure [7].

## Conclusions

Peroneal nerve dysfunction is the most prevalent compressive neuropathy in the lower extremity and should be considered in the list of possible diagnoses for patients who present with foot drop. The diagnosis is often determined by observing physical examination findings such as decreased strength, altered sensation, abnormalities in gait, and difficulties in predicting peroneal nerve dysfunction in neonates with suspected CTEV. Motor NCS and EMG also play a role in confirming the diagnosis. Our case report highlights the potential of surgical interventions to improve peroneal nerve dysfunction.
